# Temporal trends of inpatient oral penicillin challenges in a U.S. veteran cohort with a recorded penicillin allergy

**DOI:** 10.1017/ash.2025.10205

**Published:** 2025-11-03

**Authors:** Reuben J. Arasaratnam, Christine Vu, Carlos A. Alvarez

**Affiliations:** 1 Veterans Affairs North Texas Health Care System and University of Texas Southwestern Medical Center, Dallas, TX, USA; 2 Department of Pharmacy Practice and Center for Excellence in Real World Evidence, Texas Tech University Health Science Center, Dallas, TX, USA

## Abstract

In a U.S. veteran cohort (2014 – 2024) with a listed penicillin allergy, we studied the characteristics of 616 veterans that underwent an inpatient oral penicillin challenge. Notably, almost half of these challenges occurred in 2023 and 2024, suggesting recent uptake of this penicillin allergy evaluation modality within the Veterans Health Administration.

## Introduction

Unverified penicillin allergy labels are associated with several negative consequences including suboptimal antimicrobial use, development of methicillin-resistant *Staphylococcus aureus* and *Clostridioides difficile* infections,^1^ increased all-cause mortality,^2^ and a heavier economic burden on health systems.^3^ In the United States, limited access to allergists and penicillin skin testing^4^ remains a challenge in addressing unverified penicillin allergies. Direct oral penicillin challenge (DOC) has emerged as a safe and effective means of removing false penicillin allergies in low-risk patients^5^ and can be performed by nonallergists in a variety of settings.

Like other healthcare systems, variable access to allergy services/penicillin skin testing exists in the Veterans Health Administration (VHA),^6^ reinforcing the importance of DOC. However, the uptake of DOC to evaluate penicillin allergy across the VHA is not known. Thus, we sought to assess the temporal trends and associated characteristics of oral penicillin challenges administered to a U.S. veteran cohort with a listed penicillin allergy from 2014 to 2024.

## Methods

### Study design

We conducted this analysis using a retrospective cohort of U.S. veterans (≥ 18 yr of age) with a listed penicillin allergy between calendar year 2014 – 2024 who received regular VA care as defined by a) at least two outpatient visits or one inpatient in a single year or b) one outpatient visit and one prescription fill in a single year between the calendar years 2014 – 2024. We extracted patient data from the VA Corporate Data Warehouse (CDW) including demographics, comorbidities, and details on penicillin allergy listing. The index date was defined as the patient’s earliest inpatient or outpatient visit from 2014 – 2024. We defined a listed penicillin allergy as allergy to penicillin, ampicillin (including ampicillin-sulbactam), amoxicillin (including amoxicillin-clavulanate), methicillin, dicloxacillin, oxacillin, nafcillin, piperacillin (including piperacillin-tazobactam), carbenicillin or ticarcillin (including ticarcillin-clavulanate).

### Definition of cohort undergoing inpatient oral penicillin challenges

We selected those within our original cohort who a) received one or two doses only of amoxicillin, penicillin V potassium or amoxicillin-clavulanate based on the inpatient Bar Code Medication Administration (BCMA), b) received an oral penicillin *after* their original penicillin allergy entry listing and c) had an amendment of their drug allergy listing on or within 7 days of the oral penicillin administration. Additional data were collected on the calendar year and VA station in which the oral penicillin was administered. We utilized skin testing Current Procedural Terminology codes 95018 and 95004 reported the same admission as the oral penicillin challenge to estimate the proportion of direct oral challenges that followed penicillin skin testing. Using a keyword approach, we performed a text mining analysis of the drug allergy module before and after the oral penicillin challenge to ascertain changes in the frequency of comments related to successful or unsuccessful DOC. Descriptive statistics were used to summarize the cohort.

### Patient consent statement

The study was approved by the VA North Texas Health Care System Institutional Review Board. A waiver of informed consent was granted.

## Results

### Temporal trends in oral penicillin challenges across health system

**Figure 1.**
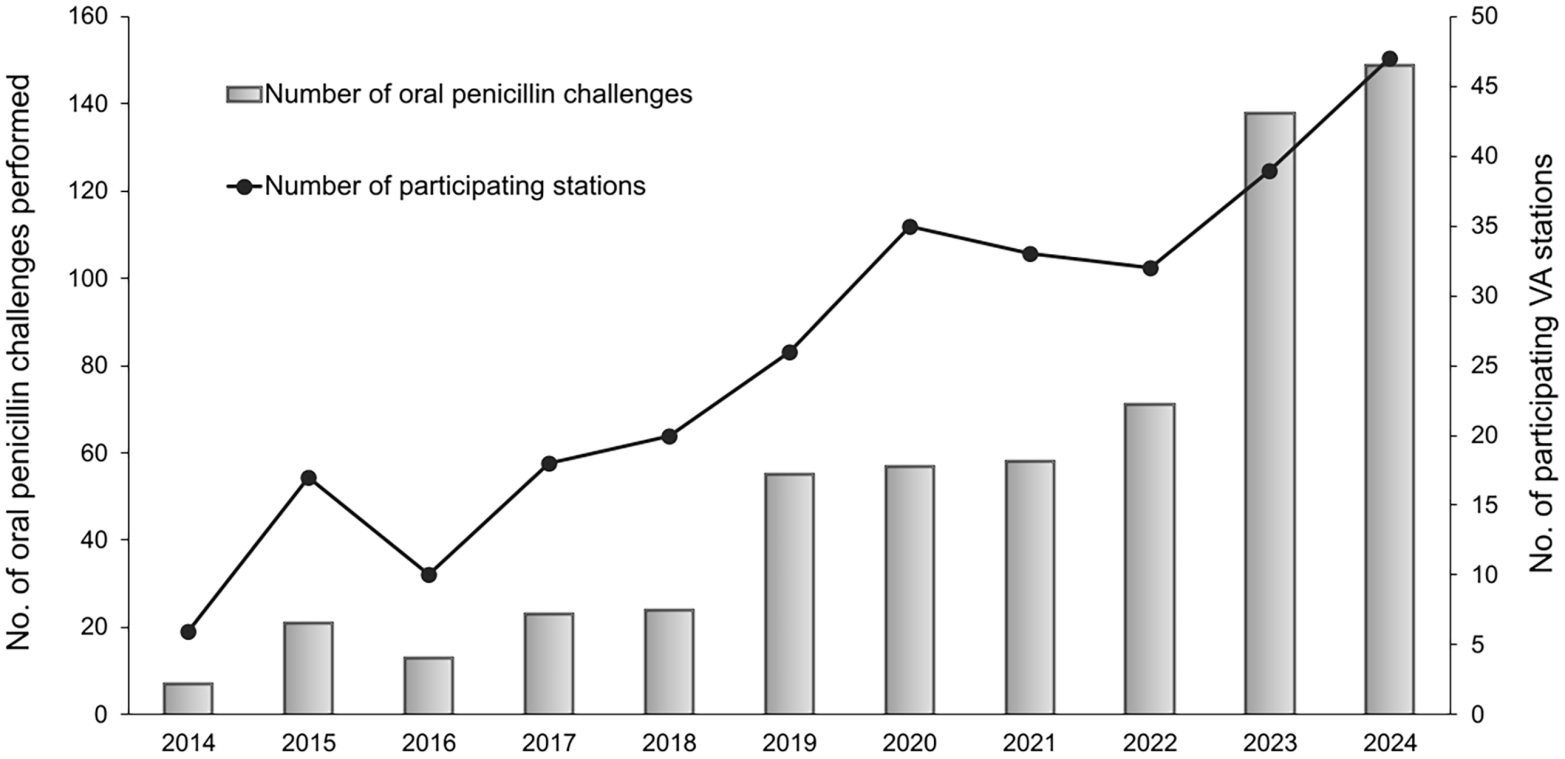
Temporal trends of oral penicillin challenges in a U.S. Veteran cohort from 2014 – 2024. The Figure shows both the number of oral penicillin challenges and participating VA stations administering challenges for individual calendar years spanning 2014 to 2024. Participating VA stations were defined as those administering a minimum of one oral penicillin challenge in a given calendar year.

From a cohort of 343,225 U.S. veterans, we identified 616 patients who underwent an oral penicillin challenge across 88 unique VA stations from 2014 to 2024. The total number of oral penicillin challenges administered per calendar year increased from 7 in 2014 to 149 in 2024, coincident with an increase in the number of VA stations administering oral penicillin challenges per year from 6 in 2014 to 47 in 2024 (Figure 1). The median number of oral penicillin challenges performed per unique VA station between 2014 – 2024 was 4 (range 1 – 38). Over 46% (*n* = 287) of challenges were performed in the calendar years 2023 and 2024.

### Characteristics of patients undergoing oral penicillin challenges

Of the 616 patients who underwent an oral penicillin challenge, the majority were male (*n* = 562, 91%), White (*n* = 462, 75%), with an average age of 60 years (Table 1). The mean Charlson Comorbidity Index was 1.4 with chronic pulmonary disease and diabetes mellitus constituting the most frequent comorbidities. Allergy to penicillin was the most frequently listed penicillin-class antibiotic listed in the drug allergy module (91%) with an average duration of penicillin allergy documentation preceding challenge of almost 10 years. Cutaneous reactions including urticaria were the most frequently documented historical reaction occurring in almost a quarter of patients.

**Table 1.** Characteristics of patients undergoing oral penicillin challenge

Characteristic	N = 616
Age, mean (SD), y	60 (12.5)
Sex, No. (%)	
Female	54 (8.8%)
Male	562 (91.2%)
Race and ethnicity, No. (%)	
White	462 (75%)
Black or African American	99 (16.1%)
American Indian or Alaska Native	5 (.8%)
Asian	1 (.1%)
Native Hawaiian or Other Pacific Island	6 (1%)
Missing/unknown	42 (6.8%)
Multiracial	1 (0.2%)
Charlson Comorbidity Index, mean (SD)	1.4 (1.7)
Charlson Comorbidity Index components, No. (%)	
AIDS/HIV	6 (1%)
Any malignancy	50 (8.1%)
Cerebrovascular disease	45 (7.3%)
Chronic pulmonary disease	123 (20%)
Congestive heart failure	40 (6.5%)
Diabetes (with and without chronic complications)	290 (37.1%)
Hemiplegia	15 (2.4%)
Liver disease	25 (4.0 %)
Myocardial infarction	13 (2.1%)
Peptic ulcer disease	3 (.5%)
Peripheral vascular disease	48 (7.8%)
Renal disease	44 (7.1)
Type of penicillin-class antibiotic allergy documented in EHR^a^	
Penicillin	558 (90.6%)
Ampicillin	6 (1%)
Amoxicillin	48 (7.8%)
Dicloxacillin	1 (.1%)
Oxacillin	1 (.1%)
Piperacillin	3 (.5%)
Concurrent cephalosporin allergy documented EHR	21 (3.4%)
Concurrent sulfonamide antibiotic allergy documented in EHR	8(1.3%)
Historical Penicillin Allergy No. (%) /Observed Penicillin Allergy No. (%)	605 (98.2%)/11(1.8%)
Average years penicillin allergy label was present at the time of challenge, mean (SD)	9.8 (8.1)
Reaction classification based on provider entry No. (%)^b^	
Anaphylaxis	17 (2.9%)
Angioedema	3 (0.5%)
Swelling	39 (6.6%)
Oropharynx and/or throat symptom manifestation	14 (2.4%)
Respiratory manifestation	20 (3.4%)
Cutaneous manifestation	147 (24.7%)
Gastrointestinal manifestation	10 (1.7%)
Neurological manifestation	3 (0.5%)
Unknown	49 (8.2%)

a Categories are not mutually exclusive as patient may have more than one penicillin-class allergy listed; percentages add to more than 100%

b 21 out of 616 did not have any reaction classification documented resulting in a denominator of 595 from which these reactions classifications are reported. The reaction categories reported are not mutually exclusive as patients may report more than one reaction to a penicillin class antibiotic.

### Characteristics of oral penicillin challenges

Amoxicillin was the most frequent penicillin used in the challenge (*n* = 426, 69%), followed by amoxicillin-clavulanate (*n* = 180, 29.2 %) and oral penicillin V potassium (*n* = 10, 1.6%) (Supplementary Table 1). A total of 4.7% of the patients undergoing oral challenge (*n* = 29) had utilization of CPT codes suggestive of penicillin skin testing associated with the oral challenge. We noted an increase in documentations surrounding penicillin allergy status following the oral penicillin challenge specifically related to descriptors supporting successful DOC including “tolerance” and “no reaction.” (Supplementary Table 1).

## Discussion

In this study of a U.S. veteran cohort with listed penicillin allergies from 2014 to 2024, we observed a total of 616 oral penicillin challenges across 88 unique VA stations that increased over time, with almost half of these challenges (*n* = 287) occurring in calendar years 2023 and 2024. This was coincident with a rise in VA stations performing oral penicillin challenges, suggesting growing adoption of DOC in the inpatient setting to evaluate penicillin allergy status across the VHA.

Several factors may account for these trends. These include the impact of the COVID-19 pandemic resulting in lower than anticipated oral penicillin challenges during the calendar years 2020 – 2022. Conversely the increase in oral penicillin challenges seen in 2023 and 2024 may be related to a pharmacy-led expansion of penicillin allergy evaluation services launched in the VHA in 2022,^7^ U.S national guidance promoting penicillin allergy evaluation^8^ published in 2022 and healthcare provider educational efforts from national allergy and antimicrobial stewardship societies in the last 3 years.^9^

Although the safety and effectiveness of DOC for penicillin allergy removal is well established, barriers to implementation have also been described particularly in cohorts such as ours composed of older adults with complex comorbidities^10^—these include provider, patient, and family member fear of allergic reaction, lack of ownership of the delabeling process, and insufficient time and staffing resources. Consistent with this, we found high variability in the numbers of oral penicillin challenges administered by VA facilities over the study period and suggest this as an area for future study.

We recognize the limitations in this report including the lack of individual direct chart verification regarding allergy listing and outcomes of oral penicillin challenges. We also acknowledge that our reported numbers of oral penicillin challenges likely underestimate the total oral penicillin challenges performed across the VHA given our focus on an inpatient cohort actively engaged in VA care and oral penicillin challenges only recorded through the BCMA. Lastly, our analysis does not capture veterans that may have undergone oral penicillin challenges at non-VA healthcare facilities and through VA Community Care.

In conclusion, this is the first VHA systemwide analysis to report a progressive increase in use of oral penicillin challenges in a U.S. veteran cohort with a listed penicillin allergy concomitant with increased VA facility participation. These findings are consistent with growing interest and active implementation of DOC as a penicillin allergy evaluation strategy across the VHA. Further study is warranted to assess the drivers of variability of implementing DOC among VA facilities.

## Supporting information

10.1017/ash.2025.10205.sm001 Arasaratnam et al. supplementary material Arasaratnam et al. supplementary material

## Supplementary material

The supplementary material for this article can be found at https://doi.org/10.1017/ash.2025.10205.

## References

[1] Blumenthal KG , Lu N , Zhang Y , Li Y , Walensky RP , Choi HK. Risk of meticillin resistant *Staphylococcus aureus* and *Clostridium difficile* in patients with a documented Penicillin allergy: population based matched cohort study. BMJ 2018;361:k2400. 29950489 10.1136/bmj.k2400 PMC6019853

[2] Blumenthal KG , Lu N , Zhang Y , Walensky RP , Choi HK. Recorded Penicillin allergy and risk of mortality: a population-based matched cohort study. J Gen Intern Med. 2019;34:1685–1687. 31011962 10.1007/s11606-019-04991-y PMC6712108

[3] Mattingly TJ , 2nd Fulton, A , Lumish RA , et al. The cost of self-reported Penicillin allergy: a systematic review. J Allergy Clin Immunol Pract 2018;6:1649–1654 e1644. 29355644 10.1016/j.jaip.2017.12.033

[4] Mancini CM , Fu X , Zhang Y , et al. Penicillin allergy evaluation access: a national survey. Clin Infect Dis. 2020;71:2972–2975. 32421192 10.1093/cid/ciaa567 PMC7947974

[5] Copaescu AM , Vogrin S , James F , et al. Efficacy of a clinical decision rule to enable direct oral challenge in patients with low-risk Penicillin allergy: the PALACE randomized clinical trial. JAMA Intern Med. 2023;183:944–952. 37459086 10.1001/jamainternmed.2023.2986 PMC10352926

[6] Kouma MA , Guastadisegni JM , Yang L , Maxwell DN , Storey DF , Arasaratnam RJ. Challenges and opportunities related to penicillin allergy in the veterans health administration: a narrative review. Antimicrob Steward Healthc Epidemiol 2023;3:e174. 38028897 10.1017/ash.2023.448 PMC10644167

[7] Allergy to Beta Lactam Evaluation (ABLE). VA Diffusion Marketplace. https://marketplace.va.gov/innovations/beta-lactam-allergy-assessment-saving-lives-one-assessment-at-a-time. Accessed 8 July, 2025.

[8] Khan DA , Banerji A , Blumenthal KG , et al. Drug allergy: a 2022 practice parameter update. J Allergy Clin Immunol 2022;150:1333–1393. 36122788 10.1016/j.jaci.2022.08.028

[9] American Academy of Allergy Asthma, and Immunology. Penicillin Allergy Center 2025. https://education.aaaai.org/penicillin-allergy-center/penicillin. Accessed 8 July 2025.

[10] Gillespie C , Sitter K , McConeghy KW , et al. Facilitators and barriers to verifying Penicillin allergies in a veteran nursing home population. J Allergy Clin Immunol Pract 2023;11:2848–2854 e2843. 37352930 10.1016/j.jaip.2023.06.023

