# Supplementary material for: Temporal trends of inpatient oral penicillin challenges in a U.S. veteran cohort with a recorded penicillin allergy
Source: Antimicrob Steward Healthc Epidemiol. 2025 Nov 3;5(1):e297. doi: 10.1017/ash.2025.10205 (PMC12616559; doi:10.1017/ash.2025.10205)
Supplement: Arasaratnam et al. supplementary material [file S2732494X25102052sup001.docx]

**Supplementary Table 1. Characteristics of oral penicillin challenges performed across VA stations in a U.S. veteran cohort with a recorded penicillin allergy**

| Characteristic | N = 616 |
| --- | --- |
| Total number of unique VA stations involved in oral penicillin challenges | 88 |
| Median number of challenges performed per facility from 2014-2024 (range) | 4 (1-38) |
| Oral penicillin used in challenge No. (%) |  |
| Amoxicillin po^a^ | 426 (69.2%) |
| Amoxicillin-clavulanate | 180 (29.2%) |
| Penicillin V potassium | 10 (1.6%) |
| Two doses of oral penicillin administered during challenge^b^ No. (%) | 178 (28.9%) |
| Penicillin skin testing CPT codes entered within 7 days of oral challenge No. (%) | 29 (4.7 %) |
|  |  |
| Comments entered in the drug allergy module^c^ | Before/after oral penicillin challenge  No. (%) |
| “Tolerate, tolerated, tolerance, tolerating” | 33 (5.6%) / 233 (39.1%) |
| “Test, tested, testing” | 12 (2.0%) / 120 (20.2%) |
| “Challenge” | 3 (0.5%) / 295 (49.6%) |
| “Remove, removed” | 1 (0.2%) / 104(17.5%) |
| “No reaction, not reactive, absence of reaction” | 4 (0.7%) / 54 (9.1%) |
| “Fail, failed, not removed, did not tolerate, did not pass” | 0 (0%) / 0(0%) |

^a^Five of these patients had amoxicillin for the first dose and then amoxicillin-clavulanate for their second dose.
^b^Of the patients receiving two doses of an oral penicillin (n=178), 89 received two doses of amoxicillin-clavulanate, 83 received two doses of amoxicillin, 5 received amoxicillin followed by amoxicillin-clavulanate, and 1 patient received two doses of oral penicillin V potassium.
^c^A total of 1621 comments were entered in 595 patients. The numbers and percentages reported utilized a denominator of 595. Comment categories reported are not mutually exclusive.
